# Deregulated microRNAs Involved in Prostate Cancer Aggressiveness and Treatment Resistance Mechanisms

**DOI:** 10.3390/cancers15123140

**Published:** 2023-06-10

**Authors:** Himali Gujrati, Siyoung Ha, Bi-Dar Wang

**Affiliations:** 1 Department of Pharmaceutical Sciences, University of Maryland Eastern Shore School of Pharmacy, Princess Anne, MD 21853, USA; 2 Hormone Related Cancers Program, University of Maryland Greenebaum Comprehensive Cancer Center, Baltimore, MD 21201, USA

**Keywords:** microRNA, CRPC, AR, PTEN, treatment resistance, splicing mechanisms, diagnostic and prognostic biomarkers, miRNA-based therapy

## Abstract

**Simple Summary:**

Accumulating studies have highlighted the critical roles of microRNAs in tumorigenesis and cancer treatment responses. Here, we systematically review the scientific publications describing the roles of microRNAs in the development and progression of prostate cancer. Numerous studies have demonstrated that microRNAs target and regulate critical genes involved in prostate cancer aggressiveness and drug resistance. However, the molecular mechanisms underlying microRNA involvement in the advanced and treatment-resistant prostate cancers remain unclear. This review aims to highlight the current understanding and knowledge gap related to the deregulation of microRNAs in prostate cancer diseases. Furthermore, we summarize the promising progress on the development of microRNA-based diagnostic/prognostic biomarkers and therapies for prostate cancer.

**Abstract:**

Prostate cancer (PCa) is the most frequently diagnosed cancer and the second leading cause of cancer deaths among American men. Complex genetic and epigenetic mechanisms are involved in the development and progression of PCa. MicroRNAs (miRNAs) are short noncoding RNAs that regulate protein expression at the post-transcriptional level by targeting mRNAs for degradation or inhibiting protein translation. In the past two decades, the field of miRNA research has rapidly expanded, and emerging evidence has revealed miRNA dysfunction to be an important epigenetic mechanism underlying a wide range of diseases, including cancers. This review article focuses on understanding the functional roles and molecular mechanisms of deregulated miRNAs in PCa aggressiveness and drug resistance based on the existing literature. Specifically, the miRNAs differentially expressed (upregulated or downregulated) in PCa vs. normal tissues, advanced vs. low-grade PCa, and treatment-responsive vs. non-responsive PCa are discussed. In particular, the oncogenic and tumor-suppressive miRNAs involved in the regulation of (1) the synthesis of the androgen receptor (AR) and its AR-V7 splice variant, (2) PTEN expression and PTEN-mediated signaling, (3) RNA splicing mechanisms, (4) chemo- and hormone-therapy resistance, and (5) racial disparities in PCa are discussed and summarized. We further provide an overview of the current advances and challenges of miRNA-based biomarkers and therapeutics in clinical practice for PCa diagnosis/prognosis and treatment.

## 1. Introduction

MicroRNAs (miRNAs) are small single-stranded, noncoding RNAs approximately 21–23 nucleotides in length. The first discovered miRNA, Lin-4, was found in *Caenorhabditis elegans* by Ambros’s group in 1993 [1], followed by the discovery of let-7 in 2000, which further led to similar findings in other species and humans [2]. In human cells, the biogenesis of miRNAs follows events in the nucleus and cytoplasm. After the initial capping and polyadenylation of primary miRNAs by RNA polymerase II (Pol II), pri-miRNAs are formed. The Drosha/DGCR8 heterodimer microprocessor further processes and crops pri-miRNAs into hairpin-structured pre-miRNAs. For the majority of miRNAs, transcription is performed by RNA polymerase II; however, some miRNAs with Alu sequence repeats are transcribed by Pol III [3]. The 364 kDa Drosha microprocessor attaches to the junction between ssRNAs and dsRNAs and two DGCR8 molecules that help to bind and stabilize Drosha [4], followed by the export of the pre-miRNAs to the cytoplasm through exportin 5 (XPO5)/Ran-GTP. In the cytoplasm, Dicer (an RNase III enzyme), along with the cofactors TAR RNA-binding protein 2 (TRBP2) and the protein activator of PKR (PACT), cleaves pre-miRNAs into small miRNA duplexes [5]. Dicer further promotes duplex unwinding through its helicase activity, resulting in one miRNA strand acting as a guide strand that complexes with the RNA-induced silencing complex (RISC). After forming the miRNA-RISC complex, it recognizes the imperfectly matched/complementary target sequences of pre-mRNAs with the help of the Argonaute (Ago) protein [6]. The primary role of these small noncoding RNAs is to recognize Watson–Crick complementary binding sites of the 3′-untranslated regions (3′-UTRs) of mRNAs and regulate their stability. While mature miRNAs are synthesized in the cytoplasmic region, reports have suggested that up to 75% of mature miRNAs can be found in both the cytoplasm and the nucleus. Apart from their main role as post-transcriptional gene regulators, miRNAs are also associated with circular RNA (circRNA), long noncoding RNAs (lncRNAs), and pseudogenes to encourage miRNA suppression or enhance competition for binding sites of miRNAs [7]. A single miRNA can concurrently target multiple mRNAs, affecting various biological processes, such as cell proliferation, cell growth, tissue differentiation, apoptosis, and embryonic development [8]. Changes in the gene expression profiles of miRNAs may influence the expression/regulation of miRNA target genes, leading to alterations in cell homeostasis [7]. The dysregulation/alteration of miRNAs is one of the crucial causes of human diseases, including cancer [9]. Accumulating studies have suggested that the deregulation of miRNAs and miRNA-mediated genes are involved in the pathogenesis of various cancers, including prostate, lung, breast, colon, head and neck cancer, liver, thyroid, endometrial, brain, ovarian, and hematologic cancers [10,11,12].

Prostate cancer (PCa) is the most frequently diagnosed cancer and the second leading cause of cancer deaths among American men [13]. Despite its prevalence, the genetic/epigenetic mechanisms underlying PCa aggressiveness and drug resistance remain elusive. Various clinical criteria, including prostate-specific antigen (PSA) levels, imaging diagnostics, and histopathological scores (such as Gleason scores), are commonly utilized as diagnosis/screening or stratification parameters for PCa [14]. However, given the limitation of these procedures in precision detection and prediction, additional diagnostic and prognostic biomarkers are needed to accurately stratify the PCa disease. In recent years, emerging data have suggested the deregulation of miRNAs as a critical epigenetic mechanism involved in the development and progression of human PCa [15]. To date, more than 50 miRNAs have been implicated in PCa disease [16]. Accumulating studies have provided convincing evidence that deregulated miRNAs are actively involved in the initiation, progression, and metastasis of PCa [17,18,19]. Specifically, differential miRNA expression signatures were identified between benign and malignant PCa [20], and deregulated miRNAs are associated with PCa development, invasion, migration, and metastasis [18,21]. Drug resistance is a leading cause of treatment failure and mortality. However, effective therapies for reversing/overcoming PCa drug resistance still remain a clinical challenge. Several mechanisms, including drug efflux, alterations in signaling, epigenetic deregulation, alternative splicing, autophagy, tumor heterogeneity, and the tumor microenvironment, have been associated with PCa drug resistance [22,23,24,25]. Notably, miRNAs are involved in the regulation of multiple key signaling pathways in PCa progression, such as androgen receptor (AR) signaling [26,27], PTEN/PI3K/AKT signaling [28], p53 signaling [29], Wnt/β-catenin signaling [30], and autophagy [31] pathways. Moreover, miRNA expression profiles are altered in response to chemotherapy, radiotherapy, and androgen deprivation therapy in PCa [32,33]. Therefore, the development of miRNA-based therapies specifically targeting aberrant signaling (AR, PTEN/PI3K/AKT, autophagy signaling, etc.) and/or alternative splicing mechanisms may further lead to novel therapeutics for treatment-resistant PCa. 

For this review, we systematically searched the literature for studies that address the deregulation of miRNAs in PCa disease. An overview of miRNAs that are downregulated (i.e., tumor-suppressive miRNAs) and upregulated (i.e., oncogenic miRNAs) is summarized and discussed. In addition, deregulated miRNAs involved in the synthesis of AR and AR isoforms, PTEN loss and/or downregulation, splicing machinery, treatment failures, and racial disparities are discussed and summarized. Finally, the current applications, limitations, and future perspectives of miRNA-based biomarkers and therapies are discussed.

## 2. miRNA Deregulation in Prostate Cancer: An Overview

In cancers, aberrantly expressed miRNAs function as either tumor-suppressive or oncogenic miRNAs [5]. The dysregulation of miRNAs, therefore, accounts for one of the mechanisms underlying the development and progression of PCa and other cancers [11]. 

Let-7, as mentioned above, was first discovered in *C. elegans* and was found to be downregulated in various cancers, and it acts as a tumor suppressor by targeting several oncogenes [34]. Previous studies have shown that decreased levels of let-7 in lung cancer directly increased the expression of the oncogene *RAS*. Conversely, restoring let-7 levels in hepatic and colon cancer restricts the growth and proliferation of cancerous cells [35]. The downregulation of let-7a has been demonstrated in several studies. For example, Guanlin et al. showed a negative correlation between the expression levels of CC chemokine receptor 7 (CCR7) and let-7a in PCa. The inhibition of let-7a resulted in the upregulation of CCR7, thereby enhancing CCR7 binding to its ligands (CCRL19 and CCRL21) and triggering the activation of MAPK-mediated EMT signaling in PCa. This hyperactivation of CCR7/MAPK/EMT signaling (due to the downregulation of let-7a) further promotes PCa cell invasion [36]. In castration-resistant prostate cancer (CRPC), let-7c is downregulated. Upon transfection of let-7c, cell growth and clonogenicity were inhibited in PCa [37]. 

MiR-1 is known to be suppressed in many cancers, including PCa, hepatocarcinoma, chordoma, and esophageal squamous cell carcinoma [38]. It has been shown that miR-1 is downregulated in PCa cells when compared to normal human prostate epithelial cells. MiR-1 is able to reduce cell viability and proliferation by targeting the c-Met/AKT/mTOR signaling pathway in PC-3 cells [38]. The luciferase assay further confirmed *MET* and *KRAS* (encoding c-Met and K-Ras, respectively) to be direct targets of miR-1 [38,39]. The inhibition of miR-1 using a miR-1 antagomir promoted cell proliferation in PC-3 cells [38]. In contrast, the overexpression of miR-1 inhibits cell proliferation, invasion, migration, and epithelial–mesenchymal transition (EMT) in PCa cells [38,39] and also suppresses tumorigenesis in xenograft PCa mouse models [39]. 

Studies have suggested that miR-29b and miR-30c play important roles in regulating cellular functions such as differentiation, proliferation, and apoptosis in cancers. RT-qPCR results have shown that the expression levels of miR-30c/-29b were reduced in PCa tissues compared to non-cancerous tissues. Additionally, a negative correlation was observed between miR-30c/-29b and cancer aggressiveness. Specifically, lower expression levels of miR-30c/-29b are correlated with more lymph node metastasis, more bone metastasis, and a higher Gleason score in PCa [40]. A previous study revealed that miR-30c targets the 3′-UTR of *SRSF1* (encoding alternative splicing factor/splicing factor 2, ASF/SF2) and *E2F7* (encoding E2F Transcription Factor 7) [41]. MiR-29b targets and negatively regulates *MCL1* and *AKT2*, while AKT2 expression suppresses Bim1. It has been shown that the overexpression of miR-29 inhibits *AKT2*, thereby upregulating Bim1 and enhancing Bim-1-mediated cell apoptosis in PCa [42]. The downregulation of tumor-suppressive miR-29b was observed in various cancers, such as lung, breast, colon, gastric, and ovarian cancers, medulloblastoma, and multiple myeloma [43,44]. 

MiR-34a and miR-99b are two important tumor-suppressive miRNAs in cancers, including PCa [45,46,47]. MiR-34a belongs to the miR-34 family, along with two other miRNAs: miR-34b and miR-34c [48]. The expression level of miR-34a is higher than that of miR-34b/c in most tissues [49,50]. MiR-34a is a direct target of p53 [51] and is involved in p53-mediated apoptosis, cell cycle arrest, and senescence [52]. Additionally, miR-34a functionally regulates the cell cycle, cell proliferation, senescence, migration, and invasion by targeting multiple genes, such as *CDK4/6*, *E2F3*, *MET*, *BCL2*, *SIRT1*, *MYC*, *NOTCH1*, and *CD44* [48,53]. Previous studies have reported that miR-34a acts as a tumor suppressor and is downregulated in a wide range of cancers, such as PCa, glioma, medulloblastoma, laryngeal cancer, lung, breast cancer, ovarian cancer, bladder cancer, colon cancer, and liver cancer [54,55,56,57]. The study by Fujita et al. showed that the expression of miR-34a was reduced in PCa specimens and cell lines when compared with their normal controls [58]. 

Another tumor-suppressive miRNA, miR-99b, belongs to the miR-125a-let-7e cluster. MiR-99b-5p is frequently downregulated and was found to regulate differentiation, proliferation, invasion, and migration in PCa, cervical, breast, esophageal, gastric, and colon cancers [59,60,61,62,63,64]. Members of miR-99 were downregulated in advanced PCa compared to the normal prostate epithelium. Through the luciferase reporter assay, three direct target genes were identified: *SMARCD1*, *SMARCA5*, and *MTOR*. In addition, by increasing the levels of miR-99 family members, a reduced level of the androgen receptor (AR) was found in the C4-2 cell line. At both the mRNA and protein levels, miR-99 members reduced PSA expression [60]. Our recent studies have also confirmed that miR-99b-5p negatively regulates the expression levels of mTOR [45,65] and AR [65] and blocks the nuclear translocation of AR and mTOR [65]. The overexpression of miR-99b-5p resulted in the inhibition of cell proliferation/viability, the enhancement of cell apoptosis, and the sensitization of PCa, breast, colon, and lung cancer cells to docetaxel [65]. 

MiR-99b-5p has been shown to be a tumor-suppressive miRNA in various cancers, including PCa [66]. In PCa, a functional link between miR-133a-5p and *FUS/AR* was revealed by Zheng et al. Specifically, miR-133a-5p directly targets the 3′-UTRs of *FUS* and *AR* and inhibits the expression of *FUS* and *AR* [67]. In addition, the overexpression of miR-133a resulted in a significant reduction in the cell viability of the PCa cell lines VCaP and LNCaP. Not only *FUS* and *AR* but also the downstream targets of AR, such as *IGF1R* and *EGFR*, were negatively regulated by miR-133a. Moreover, cell proliferation is enhanced upon the inhibition of miR-133a [67]. In another study by Tang et al., miR-133a-3p was found to directly target *EGFR*, *FGFR1*, *IGF1R*, and *MET* in PCa cells. Due to the downregulation of miR-133a-3p, EGFR/PI3K/AKT signaling is upregulated in PCa [68]. 

It has been indicated that miR-134-5p plays a critical role in suppressing human cancers [69]. It affects various oncogenic signaling pathways, such as MAPK/ERK, Notch, and EGFR signaling, by targeting different genes within those pathways. When miR-134 is upregulated, it inhibits the expression of cyclin D/cyclin D2/CDK4, KRAS, EGFR, POGLUT1, and STAT5B proteins, resulting in a decrease in cell proliferation. Furthermore, miR-134 targets and inhibits *KRAS*, *NANON*, *HNF4A*, *EGFR*, *ITGB1*, and *FOXM1*, leading to the inhibition of tumor invasion and metastasis in PCa [70]. In addition, Ngalame et al. demonstrated a negative correlation between miR-134 and *RAS* oncogenes. The downregulation of miR-134 is associated with the activation of RAS/ERK and PI3K/PTEN/AKT signaling pathways in human prostate epithelial and stem cells [71]. The study by Pelka et al. further revealed the downregulation of miR-134-5p in PCa vs. BPH and demonstrated a negative correlation between miR-134-5p expression levels and Gleason scores [69].

MiR-205 serves a major function as a tumor-suppressive miRNA in cancers. MiR-205 is known to regulate cell proliferation, migration, and invasion by regulating E-cadherin through the targeting/inhibition of *ZEB1* and *ZEB2* [72]. In PCa, miR-205 is frequently downregulated and functions as a tumor-suppressive miRNA. It inhibits the expression of the androgen receptor (AR) and mitogen-activated protein kinase (MAPK). Similarly, miR-205 is downregulated and also functions as a tumor suppressor in other cancers, such as breast cancer, liver cancer, skin cancer, glioblastoma, pancreatic cancer, colorectal cancer, and renal cancer [73]. 

MiR-221-5p is a miRNA demonstrating dual functional roles. Kiener et al. explored the tumor-suppressive role of miR-221-5p in PCa. The expression level of miR-221-5p was found to be downregulated in PCa vs. normal prostate tissue, as well as in metastasis vs. primary PCa [74]. The overexpression of miR-221-5p in a PCa cell line decreased cell proliferation, migration, and colony formation. In contrast, the knockdown of miR-221-5p led to the opposite changes [74]. MiR-221-5p has also been reported to target *SOCS1* and inhibit its expression at RNA and protein levels, subsequently suppressing Ras/Raf/MEK/ERK signaling cascades in PCa [75]. Moreover, miR-221-5p targets *BMI1* and inhibits BMI protein expression, resulting in the inhibition of PCa proliferation [76]. On the other hand, miR-221 has also been found to be upregulated in bone metastatic CRPC, suggesting its role as an oncogenic miRNA. Sun et al. identified *HECTD2* and *RAB1A* as targets of miR-221. The overexpression of miR-221 inhibits *HECTD2* and *RAB1A* expression, promoting androgen independency, AR reprogramming, and CRPC progression [77]. 

Similar to miR-221-5p, miR-375-3p represents another miRNA that potentially exhibits dual functional roles. MiR-375 was found to be downregulated in PCa. It was found that miR-375 targets and inhibits *QKI5* in PCa, inhibiting QKI5-mediated gene expression in PCa [78]. In addition, miR-375 targets/inhibits *CD44* and its splice variants. The downregulation of miR-375 was observed in androgen-independent PC-3 and DU-145 [79]. Abramovic and colleagues, however, reported that miR-375-3p is a potent PCa biomarker due to its oncogenic properties. They found that miR-375-3p was highly expressed in the blood plasma of males with PCa compared to benign prostatic hyperplasia (BPH) patients. It was also suggested to be a higher-performance diagnostic marker compared to PSA [80]. 

MiR-21 has been found to be overexpressed in various types of cancers, including PCa and a wide range of other cancers [81]. With its ability to promote cell proliferation/invasion and metastasis and inhibit apoptosis, miR-21 is known as an oncogenic miRNA [82]. A previous report demonstrated that miR-21 is overexpressed in doxorubicin (DOX)-resistant PC-3 cells compared to the parental PC-3 cells. *PTEN* was identified as a direct target of miR-21. The suppression of miR-21 resulted in the upregulation of PTEN and a significant reduction in p-glycoprotein (P-gp, an MDR protein), subsequently inhibiting PI3K/AKT signaling, reversing drug resistance, and enhancing apoptosis in DOX-resistant PC-3 cells [83]. 

Studies have shown that miR-96-5p is significantly upregulated in PCa and various cancers, and it has a functional role linked to the tumor size, metastasis, and malignancy [84]. In PCa, miR-96-5p has been shown to target tumor suppressor genes *FOXO1* and *MTSS1*. The overexpression of miR-96-5p, therefore, promotes cell proliferation, colony formation, and invasion in PCa [85]. It has also been reported that EGFR induces/activates miR-96-5p expression, which in turn targets and inhibits the tumor suppressor gene *ETV6* and consequently promotes PCa cell proliferation. In addition, miR-96-5p has been demonstrated to regulate autophagy by targeting *MTOR* and *ATG7* in PCa under hypoxia [69]. 

MiR-106b functions as an oncogene, and it is upregulated in various cancers, including PCa, breast cancer, lung cancer, gastric cancer, colorectal cancer, hepatocellular carcinoma, and esophageal squamous cell carcinoma [86]. MiR-106b target genes include *TEN*, *AKT*, *CNN1*, *LARP4B*, *RUNX3*, *DAB2*, *DLC1*, *FOG2*, *REST1*, *FUT6*, *ALEX1*, and *BTG*3 [86]. In the PCa cell line LNCaP, miR-106b directly targets and inhibits *CDKN1A* (encoding p21), which leads to a G2/M cell cycle arrest. MiR-106b also regulates the expression of Ki67, MMP2, CD44, and Smad2 proteins in PCa cells [87]. 

Another miRNA that displays oncogenic properties is miR-125b. It has been reported that PCa tumor growth is significantly increased when miR-125b is overexpressed in either intact or castrated male nude mice [88]. Notably, one of the oncogenic mechanisms underlying PCa progression is mediated through the inhibition of the pro-apoptotic genes *TP53*, *PUMA*, and *BAK1* and their downstream signaling pathways by miR-125b. In contrast, the inhibition of miR-125b increases cell apoptosis in PCa cells [88].

The oncogenic miRNA miR-141-3p is overexpressed in PCa cell lines and patient samples. It has been shown that the high-level expression of miR-141-3p promotes cell proliferation, 3D spheroid formation, tumorigenesis, stemness, and chemoresistance in PCa cell lines [89]. In addition, miR-141 is involved in cell proliferation by targeting/inhibiting *PTEN*, *BRD3*, and *UBAP1*, enhancing the activation of AKT and Rb/E2F signaling pathways in nasopharyngeal carcinoma [89]. 

The deregulation of miRNAs is one of the mechanisms conferring drug resistance in cancers, thereby leading to therapeutic failures. For example, the upregulation of miR-145 has been correlated with chemoresistance in PCa and other cancers [90]. A previous study further showed that the upregulation of miR-145 resulted in the downregulation of P-gp (P-glycoprotein) and pRb (retinoblastoma protein) by targeting and inhibiting *SP1* (encoding specific protein 1) and *CDK6* (encoding cyclin-dependent kinase 6) [90]. 

MiR-182 was also found to be upregulated in PCa. The overexpression of miR-182 results in increased cell proliferation through the targeting/regulation of the expression of *NDRG1*, *GNA13*, and *BRCA1* in PCa. The study by Bai et al. showed that miR-182 acts as an oncogene and is upregulated in breast cancer, ovarian cancer, pancreatic cancer, and colorectal cancer. In PCa, miR-182 directly targets the 3′-UTR of *ST6GALNAC5* [91]. 

MiR-200c, belonging to the miR-200 family, is known to play a role in promoting tumor progression in PCa and various cancers [92]. In the study of Lin et al., miR-200c was highly expressed in PCa cell lines compared to normal prostate cells [93]. Similarly, miR-200c is highly expressed in ovarian, endometrial, and esophageal cancers, consequently enhancing tumor growth and promoting chemoresistance [92]. However, various studies have expressed contrasting roles of miR-200c. For instance, it was found that miR-200c negatively regulates the expression of ZEB1 and vimentin, displaying an inhibitory effect on EMT-mediated cell proliferation, invasion, and migration in PCa [94]. Moreover, miR-200c acts as a tumor suppressor by inhibiting *FOXF2* and *BMI1* expression, consequently inhibiting cancer invasion and migration [95]. 

It was also found that miR-222 targets and negatively regulates *CDKN1B* (encoding p27kip1), a negative regulator of cell cycle progression in PCa and many other cancers [96]. The expression levels of miR-222 were found to be negatively correlated with PCa aggressiveness. Additionally, the overexpression of miR-222 resulted in increased cell proliferation in vitro and tumorigenicity in vivo [97]. Similar to PCa, the expression level of miR-222 was significantly associated with the tumor grade in uterine cancer [98] and tamoxifen resistance in breast cancer [99]. 

All the deregulated miRNAs described above are summarized in Table 1 below.

## 3. miRNAs Involved in AR/AR-V7 Synthesis and Hormone Therapy Resistance in PCa

### 3.1. miRNAs in AR Synthesis

Male sex hormones, namely, testosterone and dihydrotestosterone (DHT), are known as androgens, and their activities are facilitated through the androgen receptor (AR). AR is located on the X chromosome and consists of three functional domains: the DNA-binding domain, the ligand-binding domain, and the N-terminal transcriptional regulating domain [108]. Belonging to the nuclear hormone receptor superfamily, AR executes its functions by targeting/binding to consensus DNA sequences in gene promoter regions. The androgen binding to AR induces conformational changes in the AR protein structure, allowing AR to engage chromatin elements and promote the transcription of its downstream target genes [109]. It is known that the dysregulation of AR is a common cause of PCa occurrence [110]. Alterations in the AR signaling axis through the modification/modulation of mRNA/protein levels of AR, AR coregulators, and AR splice variants have been shown to act as driving forces for PCa progression [111]. Recent studies have further revealed miRNA-driven technology to be an attractive molecular strategy for regulating AR activity/signaling, and it has been proven to be of importance therapeutically. MiRNAs have the ability to directly bind to the 3′-UTR of AR and post-transcriptionally inhibit AR expression by modulating transcription, translational efficiency, and regulatory networks. AR could be targeted by miRNAs through the direct and indirect targeting of AR and/or the direct and indirect targeting of AR coregulators/modulators [112]. One of the initial studies reported by Sikand et al. showed that AR is a direct target of miR-488*, which downregulates AR transcriptional activity, inhibiting the endogenous AR protein in both androgen-dependent and androgen-independent PCa cells. Their data further suggested that miR-488* also suppresses AR-V7 expression [113]. Various studies have identified direct AR-targeting miRNAs. A study by Östling et al. defined 71 unique miRNAs that regulate the protein levels of AR (19 miRNAs upregulate and 52 miRNAs downregulate the AR levels) in PCa cell lines. Among those miRNAs, 13 miRNAs (miR-34a, miR-34c, miR-135b, miR-185, miR-297, miR-299-3p, miR-371-3p, miR-421, miR-449a, miR-449b, miR-634, miR-654-5p, and miR-9) were further validated using the luciferase assay [114]. MiR-34a is frequently downregulated in PCa, which in turn upregulates AR expression. Kashat et al. observed that the overexpression of miR-34a suppressed the expression levels of AR, PSA, and Notch-1, consequently inhibiting cell growth/proliferation in various PCa cell lines. These results suggest that AR is a direct target of miR-34a [115]. Aakula et al. reported that miR-135b directly targets the 3′-UTR of AR and downregulates AR expression in PCa and might be regulated by DNA methylation and NFkB signaling [116]. Another major study by Kumar and colleagues screened 810 miRNA mimics through complementary functional assays, including the protein lysate microarray (LMA) quantification of AR and PSA protein levels, to identify miRNAs that target AR. Forty-four mimics (out of all miRNA mimics tested) altered AR transcriptional activity, among which miR-9-5p, miR-30b-3p, and miR-541-3p were identified as the most potent AR-suppressing miRNAs. The sequence analysis identified 5 miRNAs (miR-646, miR-9-5p, miR-371-3p, miR-193a-3p, and miR-488-5p, out of 44 miRNAs) with complementary seed sequences for binding to the 3′-UTRs of AR and its AR-V7 splice variant. Three miRNAs, miR-646, miR-371-3p, and miR-193a-3p, negatively regulate the protein expression of AR and AR-V7, as evident from Western blot analysis. In particular, miR-30b-3p and miR-30d-5p were classified as miRNAs directly targeting AR, where miR-30b is significantly reduced in primary PCa, while miR-30d-5p is underexpressed in metastatic CRPC [117].

### 3.2. Deregulated miRNAs Involved in AR-V7 Synthesis

**Figure 1:**
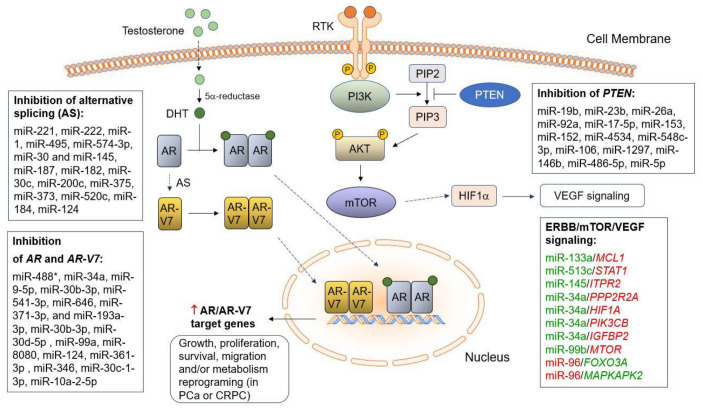
MiRNAs involved in regulation of *PTEN*, *AR*, and *AR-V7* and alternative splicing mechanisms in PCa and the deregulated miRNA–mRNA pairings involved in AA PCa disparities. The miRNAs that target *PTEN*, *AR*, and *AR-V7*, genes involved in splicing mechanisms and the alternative splicing of *TMRPSS2-ERG*, are listed. Reciprocal miRNA–mRNA pairings in AA PCa are also listed. MiRNAs/mRNAs highlighted in red and green represent upregulation and downregulation in AA vs. EA PCa, respectively. AS: alternative splicing.

Many splice variants of *AR* are known to date, and most of them are upregulated in CRPC. The most abundant *AR* variants are *AR-V1*, *AR-V7*, and *AR-V9*, with the most studied splice variant being *AR-V7* [118]. *AR-V7* is an isoform of *AR* that is alternatively spliced from *AR* mRNA. Most AR-Vs are missing portions of the COOH-terminal domain including the ligand-binding domain (LBD). The most expressed AR-V is AR-V7, known to be resistant to various drugs, including enzalutamide and abiraterone [119]. A study by Shi et al. reported on miR-124, a tumor-suppressive miRNA that is downregulated in various cancers. The study found that the 3′-UTRs of *AR-V4* and *AR-V7* transcripts are miR-124 target sites. The overexpression of miR-124 significantly downregulated AR-V7 protein levels in tumor xenografts [120]. Fletcher et al. observed that *AR-V7* mRNA was significantly decreased following transfection of a miR-361-3p mimic in the PCa cell lines 22RV1 and C4-2. Subsequently, the data suggested that miR-346 and miR-361-3p can alter the levels of the active *AR* variant and full-length *AR* [111]. Chen et al. explored the miR-30c-1-3p/miR-103a-2-5p/AR-V7 axis. The expression levels of miR-30c-1-3p/miR-103a-2-5p were downregulated in PCa cells and tumor tissues, and the overexpression of miR-30c-1-3p/miR-103a-2-5p inhibited AR-V7 expression, suppressing CRPC cell growth. It was reported that miR-30c-1-3p/miR-103a-2-5p targets the 3′-UTR of *AR-V7* and also results in the decreased expression of *AR-V7* downstream genes [121]. Figure 1 summarizes the miRNAs targeting/regulating *AR* and the *AR-V7* splice variant.

### 3.3. miRNAs Involved in Hormone Therapy Resistance of PCa

It was observed by Fujita et al. that the expression of miR-148a was downregulated in PC-3 and DU-145 cells, hormone-refractory cell lines (PC-3 and DU-145), compared to the normal prostate epithelial cell line PrEC and the hormone-sensitive cell line LNCaP. After the overexpression of miR-148a in the PC-3 cell line, the chemosensitivity to paclitaxel was increased, suggesting that miR-148a downregulation may contribute to drug resistance in PCa [122]. It was also found that miR-125b is highly expressed in androgen-independent PCa, and inhibiting miR-125b could sensitize the androgen-independent PCa to chemotherapy. MiR-125b targets *TP53*, *PUMA*, and *BAK11* [88]. Several tumor-suppressive miRNAs are downregulated in androgen-independent PCa, such as miR-31, miR-148a, miR-200b-3p, let-7c, miR-124, miR-34a/34c, miR-185, and miR-146a. The downregulation of these miRNAs leads to tumor progression, increased aggressiveness, and enhanced resistance to androgen deprivation therapy (ADT) [123]. It has been suggested that miR-216a is upregulated in androgen-sensitive LNCaP cells. *PTEN* and *TGFBR2* were predicted to be direct targets of miR-216a, and the overexpression of miR-216a was shown to promote bicalutamide resistance in LNCaP cells [124]. Additionally, miR-194 is found to be negatively correlated with the AR signaling axis. Specifically, miR-194 targets and inhibits *FOXA1*, promoting the trans-differentiation of PCa cells to neuroendocrine-like cells and enhancing enzalutamide (Enz) drug resistance in PCa [125]. Upon the loss of function of miR-101 or miR-27a, orphan nuclear receptor COUP-TFII (encoded by *NR2F2*) expression is upregulated, which in turn upregulates FOXM1 and CENPF in PCa. These two proteins are known to be important oncoproteins involved in PCa progression. Both miR-101 and miR-27a were found to be downregulated in Enz-resistant PCa cells, while the overexpression of both miRNAs improved the Enz sensitivity of resistant PCa cells [126]. MiR-30c-1-3p/miR-103a-2-5p is found to be downregulated in PCa, and the downregulation of miR-30c-1-3p/miR-103a-2-5p correlates with Enz resistance in PCa. MiR-30c-1-3p/miR-103a-2-5p targets the 3′-UTR of *AR-V7* and inhibits AR-V7 expression, thereby sensitizing CRPC to Enz [121]. Under Enz treatment, however, tumor-suppressive miR-644a negatively regulates multiple oncogenes encoding AR regulators (such as c-Myc, BCL-XL, and BCL-2). Moreover, miR-644a simultaneously inhibits the expression of *MYC*, *AKT*, *IGF1R*, and *GAPDH*, which in turn inhibits the Warburg effect in tumor cells and increases the Enz sensitivity of CRPC [127]. 

One of the mechanisms underlying androgen-induced PCa has been linked to chromosome translocation/rearrangement between *TMPRSS2* (encoding transmembrane protease serine 2) and the ETS transcription factor *ERG* [128]. *TMPRSS2-ERG* expression is detected during tumor initiation/progression in 50–60% of PCa [129]. Emerging data have suggested the critical roles of miRNAs in regulating *TMPRSS2-ERG* expression in PCa and the functional involvement of miRNAs in *TMPRSS2-ERG*-induced CRPC. For instance, an inverse correlation between the expression levels of miR-221 and the *TMPRSS2-ERG* fusion transcript was observed in PCa [130]. Furthermore, the upregulation of the miR-221 level has been functionally implicated in the metastasis of CRPC [77]. In addition, ERG is a direct target of miR-30 and miR-145. MiR-30 was shown to target and inhibit ERG at mRNA and protein levels. Moreover, the overexpression of miR-30 suppresses the EMT phenotype and inhibits cell invasion/migration in the PCa cell lines VCaP and PC-3 [131]. Hart et al. reported that miR-145 is downregulated in PCa. MiR-145 targets the 3′-UTR of *ERG* and inhibits the expression of ERG. In addition, the downregulation of miR-145 has been correlated with high expression levels of multiple ERG splice isoforms [132]. Furthermore, previous studies showed that miR-187 and miR-182 expression is negatively and positively correlated with TMPRSS2-ERG expression, respectively [133]. 

## 4. miRNAs Involved in Chemoresistance in PCa

Studies have shown that several miRNAs play a role in docetaxel (DCT) resistance. For instance, it has been reported that the downregulation of miR-143 levels induces DCT resistance in PCa cells [134]. Specifically, miR-143 downregulation results in the upregulation of the IGF-1 receptor (IGF-1R) and insulin receptor substrate 1 (IRS1). Following this, downstream signaling molecules such as VEGF are activated. The activation of the IGF-1R/IRS1/VEGF signaling cascade activates pro-survival pathways, thereby promoting DCT resistance [135]. On the other hand, miR-183 regulates cell survival pathways and is involved in DCT resistance in PCa cells. A previous study showed that miR-183 is a direct target of a long-coding RNA (lncRNA) CASC2, while the tumor suppressor gene *SPRY2* is a direct target of miR-183. It has been shown that CASC2 is downregulated in PCa. The low level of CASC2 causes the upregulation of miR-183, thereby inhibiting *SPRY2* expression and promoting DCT resistance in PCa [136]. Another miRNA, miR-21, has been shown to be highly expressed in DCT-resistant cells. Therefore, the knockdown of miR-21 in vitro resulted in enhanced DCT sensitivity in PCa cells. Specifically, miR-21 targets and inhibits *PDCD4* expression, consequently promoting cell proliferation and inhibiting the apoptotic capacity of DCT-resistant cells [137,138]. Other miRNAs are also known to regulate antiapoptotic proteins and play important roles in DCT sensitivity. For example, both miR-205 and miR-31 are downregulated in DCT-resistant cells. Restoring miR-205 and miR-31 therefore inhibits the antiapoptotic genes *BCLW* and *E2F6*, enhancing DCT-induced cytotoxicity. Intriguingly, the downregulation of miR-205 (due to hypermethylation of its promoter) de-represses *EZH2* expression, which in turn downregulates miR-31 expression [139,140]. This EZH2-dependent epigenetic silencing of miR-205/-31 is therefore considered an important mechanism mediating DCT resistance in PCa [141]. MiR-195 is frequently downregulated in DCT-resistant cells. Clusterin is involved in the anti-apoptotic mechanism in DCT-resistant cells, and the clusterin gene *CUL* is a direct target of miR-195. Therefore, the overexpression of miR-195 targets/inhibits *CUL* and sensitizes refractory PCa to DCT [142]. It has also been reported that miR-223-3p directly targets *FOXO3*, subsequently reducing the effect of DCT-induced apoptosis [143]. Similarly, miR-323 targets *TP73* (encoding the tumor suppressor p73) and thereby reduces the apoptosis induced by DCT, while miR-4735-3p inhibits *MEKK1* and reduces DCT-induced apoptosis in PCa cell lines and in PCa xenografts [144]. Samli et al. studied 86 miRNAs in paclitaxel-resistant cell lines, especially focusing on paclitaxel-resistant VCaP, PC-3, and DU-145. Out of 86 miRNAs, 14 were upregulated 2000–8000-fold. The expression levels of miR-100-5p, miR-125b-5p, and miR-182-5p were higher in paclitaxel-resistant VCaP and DU-145 cell lines compared to non-resistant cell lines. In the resistant VCaP cell line, miR-200b-3p was significantly overexpressed, and miR-34b-3p and miR-375 were significantly increased in the resistant DU-145 cell line. On the other hand, 39 (out of 86) miRNAs were downregulated 2- to 92-fold in resistant cell lines. In the resistant DU-145 cell line, miR-17-5p, miR-19b-3p, miR-20b-5p, miR-26b-5p, miR-374b-5p, and miR-616-3p were significantly downregulated. In addition, miR-19b-3p, miR-26b-5p, and miR-374b-5p were also downregulated in all paclitaxel-resistant VCaP, PC-3, and DU-145 cells. Western blot and RT-qPCR results showed that *LARP1* (encoding La-Related Protein1, LARP1) and *CCND1* (encoding Cyclin D1) were overexpressed (2- to 5-fold higher expression than controls) in all three paclitaxel-resistant cell lines [145]. MiR-15a and miR-16 are tumor suppressors and are frequently downregulated in PCa tissues. The locus that encodes for miR-15a and miR-16 is homozygously deleted in PCa patients, which is possibly associated with chemotherapy-refractory behavior in PCa. MiR-15a and miR-16 directly target the 3′-UTRs of *CCND1* and *WNT3A* (both are oncogenes that promote antiapoptosis), consequently inhibiting cell proliferation and cell invasion. Another tumor-suppressive miRNA, miR-128, is downregulated in PCa cells compared to normal prostate cells. Transfection of a miR-128 mimic into DU-145 and LNCaP increased their sensitivities to the chemotherapeutic drug cisplatin. In silico studies showed that miR-128 targets *ZEB1.* Upon the knockdown of miR-128, ZEB1 is then overexpressed, thereby promoting invasiveness and chemo-refractory behavior in PCa cell lines [146]. Additionally, studies have shown that miR-205 has the ability to enhance the drug efficacy of cisplatin in PCa. Specifically, miR-205 targets *RAB27A/LAMP3* and downregulates the expression of the lysosome-associated protein RAB27A/LAMP3, hence overcoming cisplatin resistance in PCa [32]. Taken together, the miRNAs involved in treatment resistance are summarized below (Table 2).

## 5. miRNAs Involved in PTEN Loss/Downregulation in Aggressive PCa

PTEN is one of the most frequently altered tumor suppressor genes in PCa [150]. The loss of PTEN function occurs in approximately 30% of primary CRPC and 50% of patients with metastatic CRPC (mCRPC) and is correlated with poor prognosis in PCa [150,151]. PTEN is a regulator of the important cell survival pathway PI3K/AKT/mTOR signaling, which is one of the most frequently activated pathways in PCa [152]. Therefore, the loss of PTEN leads to the upregulation of PI3K/AKT/mTOR signaling, contributing to advanced PCa progression and poor clinical outcomes [153]. 

Two members of the miR-23b cluster, miR-26a and miR-23b, promote the cell proliferation of PCa by targeting *PTEN* and suppressing downstream PI3K/AKT and cyclin D1 signaling [28]. The miR-17-92 gene cluster plays an oncogenic role in various malignancies, including PCa, and two members of the miR-17-92 cluster, miR-19b and miR-92a, promote the cell proliferation of PCa by inhibiting *PTEN*, resulting in the activation of AKT/mTOR signaling. In particular, based on their dominant inhibitory effect on *PTEN* levels, miR-26a and miR-92a function as oncogenic miRNAs in PCa.

The overexpression of miR-17-5p, one of the members of the miR-17-92 gene cluster, was shown to inhibit the tumor suppressor PTEN, causing tumor growth and invasion in PCa cells [152]. MiR-17-5p is the most prominent member of the miR-17-92 gene cluster, and it is essential for basic cellular processes, such as proliferation, cell cycle regulation, and apoptosis. MiR-17-5p inhibits *PTEN* and *CDKN1A* (p21 gene), leading to tumor development through increased cell circulation, proliferation, and tumor metastasis. High expression of miR-17-5p is associated with biochemical recurrence and an aggressive PCa phenotype [154].

High miR-153 expression and PTEN loss in PCa are significantly associated with higher Gleason patterns, higher Gleason scores, and higher-grade groups. The expression of miR-153 showed a significant negative correlation with PTEN expression [155]. It has been shown that increased miR-153 expression results in the loss of PTEN expression in PCa, suggesting that miR-153 can directly target/inhibit *PTEN* in prostate tumors and promote proliferative lesions [155].

One of the members of the miR-148/152 family, miR-152, is a tumor suppressor in human cancers. However, in one of the studies, miR-152 was reported to suppress the PTEN protein expression level by targeting the 3’-UTR region of *PTEN* in PCa [156,157]. MiR-4534 binds directly to *PTEN*, and miR-4534 exerts oncogenic effects in part by downregulating the tumor suppressor *PTEN* gene. The depletion of miR-4534 in PCa induces a tumor suppressor phenotype, in part through the induction of *PTEN*. In addition, *PTEN* induction by miR-4534 knockdown resulted in the upregulation of p53, p73, and p21, consequently suppressing cancer growth and progression and downregulating phospho-AKT protein, a molecule important for tumor development, cell survival, and invasion [150]. MiR-548 has potential binding sites in the 3′-UTR of *PTEN*. Furthermore, the overexpression of miR-548c-3p in PCa can suppress PTEN expression levels, and miR-548 expression levels were increased in high-grade PCa in comparison with normal tissues [158]. MiR-106b is significantly upregulated in PCa cells compared with normal prostate cells, and miRNA-106a directly targets *PTEN* in PCa. The overexpression of miR-106a has been shown to promote PCa cell growth and proliferation by inhibiting PTEN [159].

MiR-1297 is overexpressed in PCa patients and cell lines. *PTEN* is a direct target of miR-1297 through its four binding sites in the 3′-UTR. MiR-106a expression significantly reduced the activity of PTEN. Notably, PTEN levels were demonstrated to be significantly decreased in cancerous vs. normal tissues. Furthermore, the expression of pAKT and pERK was inhibited upon the overexpression of PTEN by silencing miR-1297. Therefore, the inhibition of the *PTEN* level by miR-1297 leads to enhanced cell migration and invasion in PCa [160].

The expression of miR-146b was significantly upregulated in PCa cell lines (LNCaP, DU-145) compared to the normal cell line (RWPE-1), while PTEN levels in PC-3 cells were notably decreased compared with those in LNCaP and DU-145 cells. MiR-146b targets *PTEN* and inhibits its expression. Moreover, targeting *PTEN* by miR-146b promotes tumorigenesis in PCa. Together, these findings also suggest that miR-146 negatively regulates PTEN-mediated signaling, thereby activating AKT/mTOR signaling and promoting survival, proliferation, and autophagy in PCa [161].

MiR-486-5p inhibits the PTEN/PI3K pathway, one of the most active signaling pathways in PCa. The overexpression of the miR-486-5p level has been shown to suppress the migration, invasion, and motility capacities of PCa. Therefore, a miR-468-5p mimic has been considered as a novel miRNA-targeted therapeutic agent for PCa [162]. MiR-21 is highly expressed in PCa tissues, and miR-21 was identified to target and inhibit *PTEN* in PCa cells. When a *PTEN*-expressing plasmid and miR-21 analog were co-transfected into PCa cells, it significantly increased cell viability/invasion and also increased the expression levels of Bcl-2, survivin, MMP2, and MMP9. These results suggest an effect of miR-21 on promoting PCa proliferation and invasion through the inhibition of *PTEN* [163,164]. All the deregulated miRNAs involved in the regulation of PTEN expression are summarized in Table 3 below.

## 6. Deregulated miRNAs Regulating Alternative Splicing Mechanisms in PCa

Serine/arginine-rich splicing factor 1 (SRSF1) is a proto-oncogene that is frequently overexpressed in various types of cancers [165]. Several studies have shown that the SRSF1 protein binds to the promoter regions of miR-221 and miR-222 and activates their expression [166]. It has been shown that miR-221 and miR-222 functionally target and inhibit the expression of tumor suppressor genes (such as *CDKN1B/p27* and *CDKN1C/p57*) and oncogenes (such as *BCL2L1* and *ER*) in various cancers, including glioblastoma, hepatocellular carcinoma, breast cancer, and PCa [167,168]. Several SRSF1-regulated miRNAs (such as miR-221 and miR-222) have also been shown to be dysregulated in tumor cells. Several genes encoding key tumor suppressors and oncoproteins, including p27, p57, BCL2L1, and the estrogen receptor (ER), have been identified as targets of miR-221 and miR-222. Using a genetic approach in glioblastoma, miR-221/222 was demonstrated to downregulate the cell cycle inhibitors/tumor suppressors *CDKN1B* (p27 gene) and *CDKN1C* (p57 gene), resulting in enhanced cell proliferation. In line with this, several studies have reported a similar association in other cancers, including PCa, hepatocellular carcinoma [167], and breast cancer [168]. The expression levels of miR-221 and miR-222 are significantly higher in androgen-independent PCa cell lines (LNCaP-Abl, LNCaP-104R2, and LNCaP-C4-2) compared to their parental androgen-dependent PCa cell line, LNCaP. The overexpression of miR-221/-222 has been demonstrated to suppress the DHT-mediated activation of PSA transcription in androgen-independent and -dependent PCa cells, while miR-221/-222 overexpression induces/enhances the DHT-independent growth of PCa cells. In contrast, transfection of a miR-221 antagomir significantly reduced cell growth and restored the androgen sensitivity of the CRPC cell line LNCaP-Abl. It has also been shown that the overexpression of miR-221/-222 significantly inhibits the expression of the tumor suppressor p27/kip1 [167]. Taken together, high-level expression of miR-221/-222 causes the inhibition of the tumor suppressor p27/kip1 and is associated with androgen-independent growth in CRPC.

SRSF9 is a member of the SR protein family, and SR proteins are considered potential drivers of cancer development/progression. The downregulation or loss of function of SR family proteins has been associated with reduced cell viability in ovarian cancer and PCa [169,170]. A previous study further showed that miR-1 negatively regulates the expression of the *SRSF9* transcript. The luciferase reporter assay further confirmed that the 3′-UTR of *SRSF9* is a direct target of miR-1 [171].

A previous study identified 4293 differentially expressed genes (DEGs) between tumor and normal tissues in 498 PCa patients from the TCGA database. The upregulation of splicing factors (including *SRSF2*, *SRSF5*, *SRSF7*, and *SRSF8*) was found in PCa vs. normal tissues. Chen et al. utilized gene set enrichment analysis and constructed a protein–protein interaction (PPI) network containing four transcription factors (TFs, i.e., ZBTB33, ATF3, ELK1, and YY1), five miRNAs (miR-495, miR-519e, miR-515-5p, miR- 217, and miR-433), and miRNA–mRNA pairs (218 DEGs targeted by the five miRNAs). Notably, this integrative genomic and miRNA–mRNA pairing analysis revealed *HNRNPH1* and *SRSF5* to be direct mRNA targets of miR-495 [172]. In addition, survival data analysis further revealed that the overexpression of *SRSF2*, *SRSF5*, and *HNRNPH1* is associated with poorer survival in PCa patients [172]. Taken together, these results suggest that the interplay between deregulated miRNAs and aberrant splicing mechanisms may play a critical role in PCa aggressiveness and/or treatment resistance/outcomes. 

Ceramide synthetases 1-6 (CerS 1-6) are involved in the generation of ceramides, and ceramides with distinct fatty acid chains function differently in cell growth and apoptosis [173]. It has been reported that the C16- or C24-ceramide level is elevated in the PCa cell lines LNCaP and DU-145 under androgen deprivation conditions [174,175]. Ceramides can be further metabolized into sphigosine-1-phosphate (SIP), subsequently promoting cell proliferation/growth and metastasis and conferring drug resistance. Notably, SIP inhibitors were applied to suppress ceramide-SIP signaling, subsequently overcoming drug resistance and significantly enhancing Enz-induced cell death in PCa cells [176]. A previous study showed that miR-574-3p is downregulated in clinical PCa samples and cell lines compared to their normal controls. Moreover, the underexpression of miR-574-3p has been associated with poor clinical outcomes and advanced tumor stages in PCa [177,178]. It has been revealed that miR-574-3p targets the 3′-UTR of *CERS1* and its splice variant in various cancer [179]. The knockdown of miR-574-5p has been shown to restore CerS1 and CerS2 expression, consequently suppressing cell proliferation and androgen independency in PCa, suggesting a novel therapeutic for PCa [179]. 

One of the mechanisms underlying androgen-induced PCa has been linked to chromosome translocation/rearrangement between *TMPRSS2* (encoding transmembrane protease serine 2) and the ETS transcription factor *ERG* [128]. *TMPRSS2-ERG* expression has been detected during tumor initiation/progression in 50–60% of PCa patients [129]. Emerging data have highlighted miRNA regulation in *TMPRSS2-ERG* expression in PCa, particularly in *TMPRSS2-ERG*-induced CRPC. An inverse correlation between the expression levels of miR-221 and the *TMPRSS2-ERG* fusion transcript was observed in PCa [130]. Notably, the miR-221 level was found to be upregulated in bone metastasis of CRPC [77], suggesting that the downregulation of miR-221 may also contribute to androgen resistance/independence during PCa progression. In addition, ERG is a direct target of miR-30 and miR-145, and miR-30 was shown to target *ERG* and inhibit its expression at mRNA and protein levels. Moreover, the overexpression of miR-30 resulted in a reduced EMT phenotype and reduced cell invasion/migration in the PCa cell lines VCaP and PC-3 [131]. Hart et al. reported that miR-145 is downregulated in PCa, and miR-145 expression is negatively correlated with ERG protein expression. MiR-145 targets the 3′-UTR of *ERG*, and the downregulation of miR-145 may be associated with high expression levels of multiple ERG splice isoforms [132]. In addition, previous studies have shown that miR-187 and miR-182 expression levels are negatively and positively correlated with *TMPRSS2-ERG* expression, respectively [133]. 

PCa demonstrates a higher expression level of alternative splicing factor/splicing factor 2 (ASF/SF2, also named SRSF1) when compared to normal tissues. The elevated SRSF1 level has been correlated with higher PCa pathological stages and recurrence. In contrast, the tumor-suppressive miRNA miR-30c is downregulated in PCa vs. normal cells. In addition, a luciferase reporter assay confirmed that the 3′-UTR of *SRSF1* is a direct target of miR-30c. The overexpression of miR-30c targets/inhibits *SRSF1* expression at both the mRNA and protein levels, consequently suppressing cell proliferation and promoting cell apoptosis [41]. SRSF1 has been implicated in regulating the alternative splicing of *VEGF* and *HSD17B2* pre-mRNAs in PCa. The upregulation of SRSF1 and SRPK1 (upstream regulator of SRSF1) promotes the generation of the aberrant splice variant *VEGF165b*, leading to more aggressive PCa with enhanced cancer growth [180,181,182]. *HSD172B2* exhibits a higher expression level in normal/benign tissues vs. PCa samples, and the expression levels of *HSD172B2* decrease as PCa progresses. *HSD172B2* encodes 17βHSD, a tumor suppressor preventing the conversion of precursors to DHT and inhibiting AR signaling. In PCa, the upregulation of SRSF1 promotes the aberrant splicing of *HSD172B2* pre-mRNA to produce *HSD172B2-M* (middle) and *HSD172B-S* (short) splice variants. The alternative M and S splice isoforms, lacking the domain for interacting with DHT, are bound to 17βHSD (encoded by *HSD172B2*) and promote the degradation of 17βHSD. This, in turn, reactivates AR signaling and promotes PCa progression in vitro and in vivo [183]. 

Previous studies have also highlighted the functional roles of miRNAs in the regulation of epithelial–mesenchymal transition (EMT) by mediating alternative splicing mechanisms. Quanking5 (QKI-5, encoded by *QKI5*) is an RNA-binding protein that modulates EMT-associated alternative splicing events. A previous study showed that QKI-5 is highly expressed in basal-like and claudin-low breast cancer subtypes (compared to LumA, LumB, and Her2 subtypes) and in high-Gleason-score and recurrent/metastatic PCa (compared to low-Gleason-score and primary PCa). In addition, an inverse correlation between miR-200c and *QKI5* expression levels was observed in breast cancer and PCa. Moreover, it was demonstrated that *QKI5* is directly targeted and negatively regulated by miR-200c and miR-375. In breast cancer and PCa, the downregulation of miR-200c and miR-375 resulted in the overexpression of QKI-5, in turn upregulating hundreds of QKI5-regulated genes (such as *ADD3*, *NFYA*, *MYO18A*, and *CD47*) and hence promoting cell proliferation, invasion, and migration [78]. Taken together, miR-200c/miR-375-mediated *QKI5* signaling may play a critical role in EMT-mediated alternative splicing programming in PCa. 

Full-length *CD44* exerts a tumor-suppressive function and is overexpressed in benign prostate epithelium, while *CD44* splice variants (*CD44v7-10*) exhibit pro-invasive activity and are overexpressed in PCa. On the other hand, miR-373 and miR-520c are downregulated in PCa patient samples and PCa cell lines, and miR-373 is barely expressed in androgen-independent PCa cell lines (such as PC-3 and DU-145). Luciferase assays confirmed *CD44* to be a direct target of miR-373, and the overexpression of miR-373 and miR-520c inhibits the expression of the CD44 protein level. The downregulation of miR-373/miR-520c, which inhibits full-length *CD44*, may partly explain the upregulation of pro-invasive/oncogenic *CD44v4-7* in PCa [79]. 

A previous study revealed that miR-184 targets and inhibits *SRSF1*, resulting in the aberrant splicing of *AR*. MiR-184/*SRSF1*-mediated *AR* splice variants are not responsive to DHT stimulation and thus promote PCa progression to androgen-insensitive/independent states [184]. An RNA-binding protein, PSF, has been implicated in PCa progression to CRPC. A previous study showed that the knockdown of PSF significantly decreased the expression levels of AR target genes in LNCaP and PC-3 cells. PSF CLIP analysis further identified a series of lncRNAs and miRNAs that are regulated by PSF. For example, AR-regulating miRNAs (such as miR-125b2, miR-99a, and miR-21) were significantly downregulated in PCa cells upon siPSF knockdown. RNA-seq results further confirmed that PSF-binding genes were significantly suppressed upon the knockdown of PSF in VCaP and LNCaP cells. Furthermore, higher binding of PSF to the intron regions of *AR* was revealed in androgen-independent/CRPC LTAD and 22Rv1 cells compared to androgen-sensitive LNCaP cells [185]. Taken together, these results suggested that PSF functions as an upstream regulator of AR target genes and AR-mediated miRNAs, regulating splicing-associated mechanisms and oncogenic signaling, especially in CRPC.

Several miRNAs have been implicated in regulating the generation of *AR-V7* in PCa. Shi et al. identified that miR-124 binds to the 3′-UTR of *AR-V7*. The overexpression of miR-124 resulted in a significant reduction in AR-V7 protein levels in the CRPC cell line 22Rv1 [120]. Kumar et al. performed miRNA library screening and identified 10 miRNAs that change AR-V7 activity. These 10 miRNAs (miR- 30b-3p, miR-30c-5p, miR-30d-5p, miR-488-5p, miR-9- 5p, miR-541-3p, miR-411-3p, miR-654, miR-138-5p, and miR-646) were shown to suppress AR-V7 protein levels in VCaP cells. Five miRNAs were revealed to target the coding region of *AR-V7*, while three miRNAs (miR-646, miR-371-3p, and miR-193a-3p) were found within the 3′-UTR of *AR-V7* and are able to inhibit AR-V7 protein levels in PC-3 cells [186]. In addition, an in silico study using the miRbase sequence database identified the 3′-UTR of *AR-V7* as a direct mRNA target of miR-8080 [187]. The overexpression of miR-8080 thus resulted in a significant reduction in AR-V7 protein production, suppressing cell proliferation and enhancing caspase-dependent cell apoptosis in the CRPC cell line 22Rv1 [187]. A previous study also showed that miR-124 directly targets *AR* and *AR* splice variants [188,189]. Moreover, the overexpression of miR-124 targeted *AR* and *AR* splice variants, subsequently inhibiting cell proliferation in the CRPC cell line 22Rv1 and the CRPC-Enz resistant line 22Rv1-EnzR [120]. 

## 7. Deregulated miRNAs and Differential miRNA–mRNA Regulatory Networks in PCa Disparities

The elimination of/reduction in disparities in cancer burden remains one of the overarching themes of the American Cancer Society (ACS) 2035 challenge goals [190]. A series of reports published by the ACS in the late 1980s documented large disparities in cancer burden by race and ethnicity [191,192,193]. PCa is now the most frequently diagnosed cancer and the second most common cause of cancer deaths among men in the United States [194]. Notably, African American (AA) men are 1.7 times more likely to develop these tumors and 2.3 times more likely to die from this disease compared to their European American (EA) counterparts [13]. Multiple socioeconomic factors have been associated with observed PCa health disparities, such as access to healthcare facilities, culture, education, diet, and treatment types [195,196]. However, higher mortality and recurrence were still observed in AAs after adjustment for socioeconomic status [197,198], indicating that intrinsic biological differences may also be involved in PCa disparities.

Recent genomic studies have demonstrated that genetic risk factors in multiple biological/regulatory systems are involved in PCa disparities [199,200,201,202]. Emerging evidence has further suggested the dysfunction of miRNAs and their regulatory mechanisms to be one of the genetic determinants promoting the PCa disparities between AAs and EAs. 

For instance, it has been reported that miR-26a is upregulated in AA PCa vs. EA PCa cells. Specifically, 13.3-fold and 2.8-fold increases were identified in primary AA vs. EA PCa (comparing RC77 T/E with RC92a/hTERT) and metastatic AA vs. EA PCa (comparing MDA PCa 2b with PC-3), respectively [203]. MiR-182 has been demonstrated to have higher expression levels in AA PCa compared to EA PCa cell lines and specimens. Furthermore, PCa patients with high miR-182 expression levels have significantly shorter biochemical recurrence (BCR)-free survival compared to patients expressing low levels of miR-182. Furthermore, miR-182 expression has been implicated in promoting cell survival, cell cycle progression, and cell growth/proliferation in PCa cells, especially in AA PCa cell lines (i.e., significant effects on AA PCa cell line MDA PCa 2b compared to EA PCa cell lines DU-145 and LNCaP) [204]. A miRNA profiling study identified that miR-132, miR-367b, miR-410, and miR-152 were significantly downregulated in AA PCa when compared to EA PCa cells. The downregulation of these miRNAs was reversed after PCa cells were treated with 5μM 5-aza-2′-deoxycytidine or a 5μM 5-aza-2′-deoxycytidine/100nM TSA combination, suggesting that the downregulation of miR-132, miR-367b, miR-410, and miR-152 may be the result of the hypermethylation of those miRNAs. Promoter sequencing analysis also confirmed that miR-152 (with the most consistent increase in expression after 5-aza-2′-deoxycytidine treatment) is hypermethylated in PCa cells. RT-qPCR analysis of PCa patient samples further confirmed that miR-152 was significantly downregulated in 50% of AA PCa patients, while it was decreased in only 35% of EA PCa patients. Low-level miR-152 expression has also been correlated with decreased BCR-free survival in PCa patients. In addition, a reciprocal regulatory mechanism was found between miR-152 and its target gene *DNMT1* (encoding DNA-methyltransferase 1), where miR-152 targets/inhibits *DNMT1* expression, while *DNMT1* expression also inhibits miR-152 expression in PCa cells. Moreover, transfection of a miR-152 mimic reduces cell proliferation, migration, and invasion in PCa cells [205]. MiR-214 has been shown to be downregulated in AA PCa (MDA PCa 2b) compared to EA PCa (PC-3, DU145, and LNCaP) cells. The overexpression of miR-214 inhibits cell viability/colony formation, induces cell cycle arrest, promotes cell invasion/migration and cell apoptosis, and sensitizes ibrutinib-induced cell death [206]. It has been reported that miR-130b is upregulated in AA PCa specimens and cell lines compared to their EA counterparts. In addition, elevated miR-130b expression correlates with poorer overall survival (OS) time and may serve as a prognostic biomarker for AA PCa. MiR-130b negatively regulates the expression of *CDKN1B*, *FHIT*, *TP53INP2*, *PTEN*, *FOXO1*, *KDM2A*, and *BTG1*. Among these genes, *FHIT* and *CDKN1B* were identified/validated as direct targets of miR-130b, especially in the AA PCa cell line MDA PCa 2b. Functionally, it has been demonstrated that miR-130b expression promotes cell proliferation, invasion, migration, and cell cycle progression [207]. It was found that miR-24 is downregulated in PCa compared to normal prostate tissues. In addition, miR-24 is downregulated in AA PCa compared to EA PCa. An analysis of the TCGA database revealed the enrichment of CpG islands and hypermethylation in the miR-24 promoter, resulting in the downregulation of miR-24 in PCa. The downregulation of miR-24 can be reversed by treatment with 5-aza-CdR (a DNA methylation inhibitor), especially in the AA PCa cell line MDA PCa 2b. Western blot and luciferase activity assays further confirmed that miR-24 targets and inhibits *AR*, *IGF1*, *IGFBP5*, and *VEGF* expression. In vitro functional analysis demonstrated that restoring miR-24 caused the inhibition of AR, PI3K/AKT, transcription factor, and fatty acid metabolism pathways, ultimately inducing cell apoptosis in AA PCa cells [208]. In a genomic study of early-onset PCa, several deregulated miRNAs were identified. Among these deregulated miRNAs, miR-142-5p and miR-150-5p were shown to be upregulated in AA PCa vs. EA PCa. MiR-142-5p has been implication in negatively regulating *CCND1*, *MAPK1*, and *PTEN*, while miR-150-5p was shown to target *TP53* and *CDK2*. Therefore, the upregulation of miR-142-5p and miR-150-5p contributes to PCa aggressiveness, progression, and poor clinical outcomes [209]. A miRNA profiling study revealed that miR-21 and miR-30c were downregulated in AA PCa vs. EA PCa. Both miRNAs were shown to be significantly downregulated in high-grade (GS > 8) and metastatic PCa [210]. In addition, miR-34b was demonstrated to be downregulated in AA PCa vs. EA PCa specimens and cell lines. Promoter analysis revealed a chromosomal deletion in the AA PCa line MDA PCa 2b, but not in the EA line DU-145. Luciferase activity and Western blot assays further confirmed that miR-34b targets/inhibits *AR* and *ETV1* expression. It has also been shown that the overexpression of miR-34b inhibits cell proliferation and induces cell apoptosis and cell cycle arrest [211]. Higher expression of miR-182 was found in AA PCa vs. EA PCa tissue samples and cell lines, and the high level of miR-182 correlates with shorter BCR-free survival. *PDCD4* was identified as a direct target of miR-182, and inhibiting miR-182 increases PDCD4 expression and consequently promotes cell apoptosis and cell cycle arrest in G2/M in AA PCa cells [204]. It has also been shown that miR-99b is downregulated in AA PCa vs. EA PCa, and the downregulation of miR-99b may contribute to PCa aggressiveness in AA [212]. The downregulation of miR-212 and the upregulation of the splicing factor HNRNPH1 were found in AA PCa vs. EA PCa. Moreover, HNRNPH1 was shown to be co-expressed with AR, and the HNRPNPH1/AR co-expression level is correlated with PCa disease progression. *HNRNPH* was identified as a direct target of miR-212. Therefore, the overexpression of a miR-212 mimic negatively regulates HNRNPH expression, subsequently inhibiting AR and AR-V7 expression in PCa. These results suggest that miR-212 is critically involved in the regulation of the HNRNPH1-AR/AR-V7 axis and PCa aggressiveness, especially in AA PCa [213].

The identification of miRNA–mRNA regulatory relationships through a correlation-based approach (i.e., integrating miRNA and mRNA profiling data, finding negative correlations between expression profiles of miRNAs and predicted mRNA targets) has been suggested as a more accurate way to identify critical miRNA–mRNA interactions in PCa [214,215,216]. In our previous studies, we combined the correlation-based approach with comprehensive pathway analysis to construct miRNA–mRNA regulatory networks underlying AA PCa disparities. Specifically, we identified a novel panel of reciprocal miRNA–mRNA pairings involved in AA PCa aggressiveness, including miR-133a/*MCL1* (down/up), miR-513c/*STAT1* (down/up), miR-145/*ITPR2* (down/up), miR-34a/*PPP2R2A* (down/up), miR-34a-5p/*HIF1A* (down/up), miR-34a-5p/*PIK3CB* (down/up), miR-34a-5p/*IGFBP2* (down/up), miR-99b-5p/*MTOR* (down/up), miR-96-5p/*FOXO3A* (up/down), and miR-96-5p/*MAPKAPK2* (up/down) [45,217]. These miRNA–mRNA pairings were defined as core regulatory components regulating the oncogenic pathways ERBB (EGFR/PI3K/AKT) [217], mTOR, and VEGF signaling [45], which are frequently hyperactivated in AA PCa. RT-qPCR, Western blot, and IHC assays confirmed the reciprocal expression profiles of deregulated miRNAs and their target mRNAs in patient specimens and cell line models derived from AA and EA PCa. Targeting the ERBB/mTOR/VEGF axis by a miR-34a-5p mimic, miR-99b-5p mimic, miR-133a mimic, miR-513c mimic, or miR-96-5p antagomir has successfully reduced cell proliferation/invasion, enhanced cell apoptosis, and promoted docetaxel-induced cytotoxicity in AA PCa cells [45,217]. More recently, we further found that the miR-99b-5p/nuclear mTOR (down/up) expression profile may serve as a promising prognostic biomarker in PCa, as well as in breast, colon, and lung cancers. Specifically, the downregulation of miR-99b-5p and the upregulation of nuclear mTOR are associated with high-grade PCa, breast, colon, and lung cancers. Additionally, miR-99b-5p negatively regulates mTOR and AR expression and blocks the nuclear translocation of mTOR, consequently inhibiting cell viability and enhancing docetaxel-induced cytotoxicity in PCa, breast, colon, and lung cancer cells [65]. Taken together, these reciprocal miRNA–mRNA pairings could potentially serve as precision diagnostic/prognostic biomarkers and novel drug targets for aggressive AA PCa. 

## 8. Clinical Implications of miRNAs as Diagnostic and Prognostic Biomarkers and Therapeutic Tools/Targets

Emerging data suggest that the alteration of miRNA profiles may be associated with the development and/or progression of PCa disease. Several clinical studies were initiated to evaluate whether miRNA expression levels/profiles could serve as diagnostic biomarkers and/or are associated with cancer progression and clinical outcomes. For example, the circulating miR-141 was found to be upregulated in PCa with a moderate correlation with serum PSA levels [218]. MiR-141 expression was also detected in a wide range of human cancers [219]. The potential of utilizing miR-141 and miR-375 expression levels as indicators of clinical outcomes in low-risk PCa is being investigated in a phase II clinical trial (NCT02391051). Clinical trial NCT01220427, led by Wuerzburg University Hospital in Germany, was a study to investigate whether specific miRNA expression profiles were related to high-grade PCa. The correlation of circulating miRNA expression profiles with bone and lymph node metastases in PCa patients is under investigation in a clinical trial (NCT02964351). In a clinical trial conducted by Assuta Medical Center (NCT02964351), adenocarcinoma PCa patients with high PSA values will be enrolled, and their mRNA expression profiles will be examined using Nano-string technology. This study aims to investigate whether specific miRNA expression patterns are correlated with high-PSA adenocarcinoma PCa. Several other studies focused on miRNA signatures to predict treatment responses. For instance, NCT02366494, conducted by the Medical College of Wisconsin, is a clinical trial on the utilization of serum exosomal miRNA signatures to predict patient responses to androgen deprivation therapy (ADT). NCT02471469 is a clinical trial led by Radboud University Medical Center in which the expression profile of a panel of mRNAs, miRNAs, and lncRNAs is used as an index to personalize the enzalutamide therapy. Specifically, six miRNAs (miR-21, miR-141, miR-200a, miR-200c, miR-375, and miR-3687) were selected in the panel for predicting metastatic CRPC (mCRPC) patient responses to enzalutamide [220]. Similarly, the University of Washington and NCI have been collaborating in a clinical trial (NCT01503229) to correlate the expression profiles of circulating miRNAs with the responses of mCRPC patients to abiraterone acetate, an androgen synthesis inhibitor. NCT01120236 is a phase II clinical trial led by NCI to study whether specific miRNA expression profiles are correlated with PSA responses and/or circulating tumor cell (CTC) counts after metastatic PCa patients are treated with androgen deprivation therapy and cixutumumab for 28 weeks. Besides the miRNAs in CTCs, the specific miRNA expression signatures in liquid biopsies, such as extracellular vesicles (EVs), circulating tumor DNAs (ctDNAs), and RNAs (ctRNAs) from body fluids (blood, urine, sperm, saliva, etc.), could potentially serve as novel biomarkers for PCa diagnosis and prognosis [221]. In addition, Rana et al. conducted a systematic analysis to evaluate the potential of using miRNAs as a prognostic index for biochemical recurrence (BCR). They concluded that ten miRNAs (let-7a-5p, miR-148a-3p, miR-203a-3p, miR-26b-5p, miR30a-3p, miR-30c-5p, miR-30e-3p, miR-374a-5p, miR-425-3p, and miR-582-5p) were significantly associated with BCR among PCa patients [222]. Further verification of these miRNAs in preclinical and clinical models may warrant the development of potential prognostic biomarkers in PCa.

Furthermore, miRNAs have also emerged as potential therapeutic molecules or targets in cancer due to their ability to regulate key genes involved in cancer progression and metastasis [223]. MiRNA-based therapies aim to restore or inhibit the expression of specific miRNAs to target cancer cells and inhibit tumor growth [224]. 

One approach to miRNA-based therapies is the use of miRNA mimics, which are synthetic RNA molecules that mimic endogenous miRNAs to enhance their expression [225]. These miRNA mimics are delivered to cancer cells using various delivery systems, such as viral vectors, liposomes, and nanoparticles. Once inside the cancer cells, the miRNA mimics bind to their target mRNAs and inhibit their expression, leading to the inhibition of tumor growth and metastasis [226]. On the other hand, miRNA inhibitors, also known as antimiRs, are another type of miRNA-based therapy that targets/binds endogenous miRNAs. AntimiRs are chemically modified RNA molecules that bind to endogenous miRNAs and prevent their binding to their target mRNAs, restoring the expression of target genes that have been originally inhibited by endogenous miRNAs [227]. AntimiRs can be delivered to cancer cells through delivery systems in a similar manner to delivering miRNA mimics.

Several preclinical studies have shown promising results for miRNA-based therapies in various types of cancers. For instance, the use of miR-34a mimics has been shown to inhibit tumor growth and induce apoptosis in preclinical models of lung cancer and pancreatic cancer [228,229]. In addition, the use of miR-16 inhibitors has been shown to sensitize breast cancer cells to the chemotherapeutic agent paclitaxel [230]. MiR-34a is a major tumor-suppressive miRNA targeting/regulating a wide range of genes involved in cell proliferation, apoptosis, and metastasis. MRX34 is a liposomal nanoparticle formulation of a miR-34a mimic. The miRNA-based therapy MRX34 was under investigation in a phase I clinical trial (NCT01829971) for treating advanced solid tumors. In preclinical studies, MRX34 demonstrated efficacy in a wide range of cancers, including non-small-cell lung cancer (NSCLC), liver cancer, and renal cell carcinoma [231]. The liposomal formulation of MRX34 enables the targeted delivery of miRNA to cancer cells, which reduces off-target effects and improves therapeutic efficacy. MRX34 has also shown promising results in combination with other cancer therapies, such as chemotherapy and immune checkpoint inhibitors [232]. However, despite the promising preclinical data, clinical trials of MRX34 have faced challenges in the clinical trial. In the phase I trial of MRX34 in patients with advanced solid tumors, several patients experienced serious adverse events, including immune-related adverse events and liver dysfunction, leading to the termination of the trial [232].

MRG-106 (also called Cobomarsen), developed by miRagen Therapeutics, is under investigation in a phase II clinical trial (NCT03713320). In this trial, a miR-155 inhibitor was used for the treatment of cutaneous T-cell lymphoma (CTCL). MiR-155 is an oncogenic miRNA implicated in promoting the growth and survival of CTCL [233,234]. TargomiRs containing a miR-16-based miRNA mimic were used in a phase I clinical trial (NCT02369198) for the treatment of patients with recurrent non-small-cell lung cancer (NSCLC) and malignant pleural mesothelioma (MPM). Specifically, TargomiRs are minicells containing a miR-16 mimic specifically targeting EGFR-expressing cancer cells. Previous study showed that transfection of a miR-16 mimic inhibits the growth of MPM cell lines, sensitizing MPM cells to gemcitabine and pemetrexed. Additionally, the delivery of the miR-16 mimic inhibited MPM tumor growth in preclinical models [235]. 

Although MRX34, MRG-106, and TargomiRs have not been tested in clinical trials for PCa patients, several preclinical studies have demonstrated promising therapeutic effects of miR-34a and miR-16 mimics. For instance, several translational studies of miR-34a were performed to show the efficacy of miR-34a for treating PC-3 and DU145 xenografts, paclitaxel-resistant PCa, and bone metastatic PCa models [236,237,238,239,240]. RNA-aptamer-conjugated atelocollagen was used to deliver miR-16 to treat a bone metastatic PCa model in vivo. Moreover, miR-16 was shown to inhibit multiple cell-cycle-regulated genes, such as *CDK1* and *CDK2*. The systemic delivery of miR-16 by atelocollagen demonstrated significant inhibitory efficacy in a bone metastatic PCa model [241,242]. Taken together, miRNA-based therapies have shown promising potential for treating PCa patients in future clinical trials. MiRNA-based biomarkers and therapies are summarized in Table 4 below.

## 9. Conclusions

Accumulating studies have shown that the deregulation of miRNAs may be associated with PCa pathogenesis by disrupting cell cycle control; promoting cell proliferation, survival, invasion, metastasis; and/or evading immune destruction. In this review, we systemically searched the literature and summarized the miRNAs involved in PCa development and progression. In addition, critical miRNAs implicated in the regulation of AR/AR-V7, PTEN, splicing mechanisms, and treatment resistance were highlighted in this review. Moreover, miRNAs promoting PCa disparities were highlighted and associated with PCa aggressiveness and/or drug resistance. Emerging evidence has also demonstrated a promising opportunity to use miRNAs in clinic applications for PCa. A growing body of investigations throughout the past two decades has shown that specific miRNA profiles or a subset of miRNAs may be used to characterize different tumor subtypes, correlate with the disease stages, and/or predict clinical outcomes. On the other hand, miRNA-based therapies represent a promising molecular strategy to treat cancers in combination with chemotherapy, radiation therapy, targeted therapy, and immunotherapy. Although miRNA-based biomarkers and therapies have been demonstrated to have great potential for clinical application, challenges remain to be overcome before translating these technologies into clinical practice. For instance, the methodology, study design, sample collection/processing, profiling platforms, and data analysis should be further standardized to minimize data inconsistencies between different research groups, ultimately facilitating the development of more accurate miRNA-based biomarkers for PCa. In addition, further developing more efficient delivery systems with tissue specificity, generating more stable miRNA mimics/inhibitors, and minimizing off-target effects and immune activation will warrant the development of more effective miRNA-based therapeutics in clinical practice.

## Figures and Tables

**Table 1:** Deregulated miRNAs in PCa and other cancers.

MiRNAs	Regulation in PCa	MiRNA Target Genes and Regulated Pathways in PCa	References
let-7/let-7a	Downregulation	*CCR7* MAPK/EMT signaling pathways	[35,36]
miR-1	Downregulation	*MET*, *KRAS* c-Met/AKT/mTOR signaling	[38,39]
miR-29b	Downregulation	*AKT2*, *MCL1*	[42,44]
miR-30c	Downregulation	*ASF/SF2*, *E2F7*	[40,41]
miR-34a	Downregulation	*SIRT1*, *E2F1*, *CDK6*, *Cyclin D1*, *E2F3*, *BCL2*	[55,57,58]
miR-99b	Downregulation	*MTOR*, *SMARCA5*, *SMARCD1*, *PSA*, *AR*	[59,60,62,100]
miR-133a	Downregulation	*FUS*, *AR*, *EGFR*, *FGFR1*, *IGF1R*, and *MET*	[67,68,101]
miR-134	Downregulation	*EGFR*, *cyclin D/cyclin D2/CDK4*, *KRAS*, *EGFR*, *POGLUT1*, *STAT5B*, *KRAS*, *NANOG*, *HNF4A*, *EGFR*, *ITGB1*, *FOXM1*, *HER2*, *PIK3CA*, *ITGB1*, *POGLUT1*	[69,70,102]
miR-205	Downregulation	*AR*, *MAPK*	[73]
miR-221	Downregulation Upregulation	*SOCS1*, *BMI1* Ras/Raf/MEK/ERK signaling *HECTD2*, *RAB1A*, AR reprograming in CRPC	[74,75,76,77]
miR-375	Downregulation	*QKI5*, *CD44*	[78,79,80]
miR-21	Upregulation	*PTEN*, *RECK*, *MARCKS*, *PDCD4*, *TPM1* JAK/STAT3 cascades	[82,83,103]
miR-96	Upregulation	*FOXO1*, *MTSS1*, *EGFR*, *MTOR*, *ATG7*	[69,104,105]
miR-106b	Upregulation	*TEN*, *AKT*, *CNN1*, *LARP4B*, *RUNX3*, *DAB2*, *DLC1*, *FOG2*, *REST1*, *FUT6*, *ALEX1* and *BTG*3, *CDKN1A* (p21 gene), *E2F1*, *Ki67*, *MMP2*, *CD44*, and *SMAD2*	[86]
miR-125b	Upregulation	*BAK1*, *TP53*, *PUMA*	[88,106]
miR-141	Upregulation	AKT and Rb/E2F signaling	[89,107]
miR-145	Upregulation	*SP1*, *CDK6*, *AKAP12*, *RAD51*, *MCL1*, *PAR4*, *PARP1*	[90]
miR-182	Upregulation	*NDRG1*, *GNA13*, *BRCA1*, *PDCD4*, *ST6GALNAC5*	[91]
miR-200c	Upregulation	*BRD7*, *BMI1* EMT, AKT signaling pathways	[93,94,95]
miR-222	Upregulation	*CDKN1B*, *CDKN1C*	[97]

**Table 2:** MiRNAs involved in treatment resistance. ↓: downregulated in PCa; ↑: upregulated in PCa; ADT: androgen deprivation therapy; Enz: enzalutamide; DCT: docetaxel.

MiRNA (Up or Down in PCa)	Target Gene(s)	Treatment Resistance	References
miR-148a ↓	*MSK1*	ADT resistance	[122]
miR-125b ↓	*TP53*, *PUMA*, *BAK11*	ADT resistance	[88]
miR-216a ↑	*PTEN*, *TGFBR2*	ADT resistance	[124]
miR-194 ↓	*FOXA1*	Enz resistance	[125]
miR-27a, miR-101 ↓	*NR2F2*	Enz resistance	[126]
miR-30c-1/miR-103a-2 ↑	*AR-V7*	Enz resistance	[121]
miR-644a ↓	*MYC*, *BCLXL*, *BCL2*, *AKT*, *IGF1R*	Enz resistance	[125]
miR-221 ↓	*ERG*	Androgen-independent	[130,131]
miR-145 ↓	*ERG*	Enz resistance	[132]
miR-187 ↓, miR-182 ↑	*TMPRSS2-ERG*	Enz resistance	[133]
miR-143 ↓	*IGF1R*, *IRS1*	DCT resistance	[135]
miR-183		DCT resistance	[147]
miR-21 ↑	*PDCD4*	DCT resistance	[137,138]
miR-205, miR-31 ↓	*BCLW*, *E2F6*	DCT resistance	[139,140]
miR-195 ↓	*CUL*	DCT resistance	[142]
miR-223-3p ↑	*FOXO3*	DCT resistance	[143]
miR-4735-3p ↓	*MEKK1*	DCT resistance	[144]
miR-19b-3p, miR-26b-5p, miR-374b-5p ↓	*LARP1*, *CCND1*	Paclitaxel resistance	[145]
miR-15a, miR-16 ↓	*CCND1*, *WNT3A*	Cisplatin resistance	[146]
miR-128 ↓	*ZEB1*, *BIM1*	Cisplatin resistance	[148,149]
miR-205 ↓	*RAB27A*, *LAMP3*	Cisplatin resistance	[32]

**Table 3:** MiRNAs that regulate *PTEN* expression in PCa. ↑: increased in PCa.

MiRNA	Regulation of PTEN Expression	Function	Reference
miR-19b	Downregulates *PTEN*	Cell proliferation ↑	[28]
miR-23b	Downregulates *PTEN*	Cell proliferation ↑	[28]
miR-26a	Downregulates *PTEN*	Cell proliferation ↑	[28]
miR-92a	Downregulates *PTEN*	Cell proliferation ↑	[28]
miR-17-5p	Downregulates *PTEN*	Tumor growth ↑/cell proliferation ↑/invasion ↑	[152,154]
miR-153	Downregulates *PTEN*	Higher Gleason scores	[155]
miR-152	Downregulates *PTEN*	Tumor growth ↑	[156,157]
miR-4534	Downregulates PTEN	Cell growth ↑, proliferation ↑, and progression ↑	[150]
miR-548c-3p	Downregulates *PTEN*	Higher Gleason scores	[158]
miR-106	Downregulates *PTEN*	Cell growth and proliferation ↑	[159]
miR-1297	Downregulates *PTEN*	Migration ↑, invasion ↑	[160]
miR-146b	Downregulates *PTEN*	Tumorigenesis ↑, survival ↑, proliferation ↑, AKT/mTOR signaling↑	[161]
miR-486-5p	Downregulates *PTEN*	Migration ↑, invasion ↑, motility ↑	[162]
miR-21-5p	Downregulates *PTEN*	Cell proliferation ↑, invasion ↑	[163,164]

**Table 4:** MiRNA-based biomarkers and therapies in clinical trials. ADT: androgen deprivation therapy; mCRPC: metastatic castration-resistant prostate cancer; Enz: enzalutamide; Abi: abiraterone; CTCL: cutaneous T-cell lymphoma; NSCLC: non-small-cell lung cancer.

MiRNA	Clinical Trial	Biomarker/Therapy	Status
miR-141, miR-375	NCT02391051	Prognostic biomarker for low-risk PCa	Phase II
miRNA profiling	NCT01220427	Diagnostic biomarker for high-grade PCa	Phase II
miRNA profiling	NCT02964351	Biomarker for high-PSA PCa	Recruiting
Exosomal miRNA signature	NCT02366494	Prognostic biomarker for PCa under ADT treatment	Active, not recruiting
miRNA panel	NCT02471469	Prognostic biomarker for mCRPC under Enz	Complete recruiting
miRNA profiling	NCT01503229	Prognostic biomarker for mCRPC under Abi	Phase II
miRNA profiling	NCT01120236	Prognostic biomarker for PCa under ADT/cixutumumab	Phase II
MRX34	NCT01829971	miR-34a-based therapy for multiple solid tumors	Phase I, terminated
MRG-106	NCT03713320	miR-155 inhibitor for treating CTCL	Phase II
TargomiRs	NCT02369198	miR-16-based therapy for NSCLC	Phase I
